# Expanding the Question–Persuade–Refer (QPR) Evidence Base: Youth Suicide Prevention among the Mississippi Band of Choctaw Indians

**DOI:** 10.3390/healthcare12080834

**Published:** 2024-04-15

**Authors:** John P. Bartkowski, Katherine Klee, Xiaohe Xu

**Affiliations:** 1 Department of Sociology and Demography, University of Texas at San Antonio, San Antonio, TX 78249, USA; john.bartkowski@utsa.edu (J.P.B.); xiaohe.xu@utsa.edu (X.X.); 2 Bartkowski & Associates Research Team, San Antonio, TX 78258, USA

**Keywords:** mental health, suicide, young adult, youth, adolescent, evidence-based, training, gatekeeper, native, rural

## Abstract

Youth suicide risks have been on the rise or persistently elevated for decades, and Native American communities are especially vulnerable. This study provides a promising framework for suicide prevention among underserved populations in the U.S., especially Native American communities in states lacking strong suicide prevention supports. Our investigation reports the evaluation results of the Question–Persuade–Refer (QPR) gatekeeper training program, a key component of the SAMHSA-funded Choctaw Youth Resilience Initiative (CYRI) implemented by the Mississippi Band of Choctaw Indians (MBCI). QPR trains adult gatekeepers to identify youth at risk of suicide and refer them to certified mental health service providers. Standardized QPR pre-test and post-test training surveys were administered at in-person trainings delivered to youth-serving MBCI organization leaders and staff. Statistical analyses of all survey items indicate that QPR gatekeeper trainings significantly enhanced the knowledge of prevention practices and risk identification skills for the MBCI trainees. The robust evidence of positive changes revealed in this study suggests that QPR can be an effective suicide prevention program for underserved minority communities, especially Native American populations in rural states where suicide is a persistent and leading cause of mortality.

## 1. Introduction

Suicide remains a formidable challenge across the United States, especially in Mississippi where death by suicide has persistently been a leading cause of mortality across age groups [1,2,3]. Mississippi’s age-adjusted suicide death rate in 2021 was 16.18 completed suicides per 100,000 individuals compared to the nation’s rate of 14.04 [4]. Mississippi ranks thirteenth in the United States for completed suicides among adolescents aged 15–19, with 10.4 suicide deaths per 100,000 youth [5]. Sociodemographic characteristics like race/ethnicity often play primary roles in suicidality (i.e., suicidal thoughts and behaviors). Among American Indian and Alaska Native populations, the rates of death by suicide increased from 17 per 100,000 in 2011 to 24 per 100,000 in 2020. These rates compare quite unfavorably to the overall U.S. rates of death by suicide, which have increased slightly from 12 to 14 per 100,000 [6,7]. In 2019, suicide was the second leading cause of death for youth and young adult American Indians and Alaska Natives, with the overall death rate from suicide being 20% higher for these groups than for non-Hispanic whites [8]. 

Native Americans are an important and distinctive ethnic group within the United States with a deeply rooted history of cultural assaults and continuing oppression that exacerbate high rates of mental health and substance use disorders [9]. Under-resourced mental healthcare practices further maintain these disparities. Indigenous community mental health workers hold local understandings of their community’s history, culture, and traditional views related to health and well-being. Being culturally and scientifically informed may reduce barriers to care while promoting tribal health and economic self-determination and sovereignty. Youth and adults alike have lauded the mental and physical health benefits of reconnecting youth with the tribal language and land through culture camps, hunting, fishing, and storytelling to convey the cultural significance of their tribal lands. The importance of land and place has been found in other works of qualitative research examining culturally competent approaches to American Indian healing and well-being [10,11]. Programs for mental health crisis intervention have proven successful, particularly among indigenous groups due to their inclusion of culturally relevant resources and community endorsement [12]. Our study lends credence to the influential factors of social associations and ethnic traditions on mental health support among community-centered groups. 

Several suicide prevention programs have long been introduced for adults who work with youth at risk of suicide. The Question–Persuade–Refer (QPR) Institute has created several suicide prevention training courses for a variety of suicide-related topics and trainees’ professional backgrounds [13]. QPR gatekeeper training courses use an evidence-based methodology that has been implemented in higher education, medical, and professional settings through online or in-person instruction and certification. QPR training has proven to be effective in its ability to significantly improve the attitudes, subjective norms, and perceived behavioral capacity for suicide intervention, along with the intention to intervene [14,15]. The three-pronged approach of question, persuade, and refer encourages gatekeepers (influential adults involved in youth-serving contexts) to follow this guide that can be applied in any setting with any individual. The completion of QPR gatekeeper training courses often results in increased suicide prevention knowledge, attitudes, and skills (i.e., identifying suicide warning signs, asking about suicide risk, influencing help-seeking behavior, knowing how to recommend local resources, talking about resources, accompanying a person to get help, and calling a crisis line); the courses also increase the knowledge of suicide prevention facts and an understanding of the broader suicide context [16]. The long-term effectiveness of the QPR gatekeeper program has also been observed. Applying QPR gatekeeper training in educational and religious contexts has yielded significant results, where strong training effects were noticeable after training completion [17]. QPR trainees who interact with youth on a professional basis noted increases in the knowledge of identifying risk indicators (i.e., depressive behaviors or emotional outbursts, and evidence of self-harm), self-efficacy in navigating suicide-related conversations with youth, and help-giving behaviors [18].

QPR gatekeeper training helps adults engage in youth suicide prevention even in the face of significant challenges. Adults, and even older adolescents, working with youth often navigate the emotional and mental turbulence that comes with biological changes, school challenges, and family life difficulties. Additionally, organizational approaches to suicide prevention are often linked to changes in suicide prevention attitudes, confidence, and social norms. In one study, after QPR training completion, community support personnel showed improvements in the awareness of social norms and effective techniques related to youth suicide prevention [19]. Similarly, juvenile justice and child welfare workers engaged in more suicide prevention behaviors. Trainees across professions improved in suicide prevention attitudes, though law enforcement personnel saw little to no growth. School staff with low baseline pre-test indicators showed the most improvement in suicide identification behaviors, especially if the staff were already communicating with students about suicide and distress [20,21,22]. Students in higher education have also shown significant improvements in confidence and knowledge after completing QPR gatekeeper training, with a majority being very or extremely likely to intervene the next time they would see warning signs of suicide [23,24,25,26,27,28]. Similarly, trainee education levels have been identified as influential factors in relation to training outcomes. A study of Native American and Alaska Native adults who interact with youth in Michigan used a comparative analysis of ASIST and SafeTALK, two evidence-based forms of gatekeeper training [29]. Trainees with a lower education level (high school or two years of college) benefitted the most from training materials and better retained the training content over a longer period of time. A few variables outside of trainee characteristics and the professional environment have been identified as influential factors in training outcomes. Trainee participation and, as a result, trainee reception of training materials and changes in trainee knowledge and self-efficacy are dependent on the training group size, attitudes of trainers, and perceived behavioral control before training completion [30]. 

Our study features the evaluation results of QPR gatekeeper trainings administered to Mississippi Band of Choctaw Indian (MBCI) members or affiliates who serve MBCI-supported schools and youth-serving organizations. Information examining gatekeeper suicide prevention trainings in Native American tribal settings is limited. To date, no published systematic research of which we are aware has explored the effectiveness of QPR training among tribal-supported organizations in Southern states. While pre-test and post-test evaluations have been widely used as evidence of training effectiveness, our study adds to the current research by enlisting in-person MBCI training assessments, thereby examining the potential role of tribal influences on QPR training outcomes. Our study aims to understand the level of effectiveness in QPR training outcomes for adults who work with MBCI youth. Specifically, our study examines pre-test and post-test training outcomes that include suicide prevention knowledge, self-rated efficacy in applying suicide prevention techniques, and willingness to interact with suicidal individuals. Consistent with program evaluation studies, we do not test hypotheses per se, but generally anticipate salutary effects of the intervention. We do, of course, entertain prospective results to the contrary because Native American populations have been understudied with respect to QPR. In short, our study is exploratory in nature. 

## 2. Materials and Methods

### 2.1. Research Design

A pre-test/post-test survey design was used to assess participant knowledge and perception changes before and after the QPR training was completed. Pre-test (pre-training survey) and post-test (post-training survey) responses from QPR training sessions conducted between 2020 and 2023 were analyzed for this study. The QPR Gatekeeper Training Program was conducted as part of the Mississippi Band of Choctaw Indians’ (MBCI) Choctaw Youth Resilience Initiative—Mississippi (CYRI-MS), in partnership with the Mississippi Public Health Institute, and funded by the Substance Abuse and Mental Health Services Administration (SAMHSA). The MBCI is a self-governing Native American tribe of 11,000 individuals historically facing cultural marginalization in a consistently impoverished, racially segregated state. The MBCI is also the only federally recognized tribe in the state [31]. CYRI aimed to increase the number of youth-serving organizations able to identify and work with youth at risk of suicide and increase the capacity of clinical service providers to assess and treat youth at risk of suicide. CYRI supported the implementation of several suicide prevention strategies in the Choctaw tribal community, with the QPR training program serving as a centerpiece of this initiative. A pre–post survey design using the QPR standardized evaluation tool was selected to evaluate participants’ self-rated knowledge and perceptions of suicide prevention topics in comparative analyses for before and after responses. 

The QPR pre-training and post-training surveys were developed by the Question–Persuade–Refer national program and included a range of sociodemographic and knowledge rating items. The completion of the pre–post evaluation surveys remains anonymous. Pre–post survey matching was available and used for this study. CYRI supported the QPR training and instructor certification of MBCI suicide prevention specialists to implement QPR trainings across MBCI-affiliated youth-serving organizations, including primary, secondary, and higher-education institutions. In 2021, young adults between the ages of 18 and 24 had the highest rankings of suicide (third leading cause of death) among Mississippians, with Native Americans having the second highest suicide rate (16.74) in the United States [1,32]. MBCI tribal members often reside in rural areas of Mississippi typically beset with significant health disparities. Mississippi’s rurality lends itself to a lack of healthcare access, where 61% of communities do not have an adequate amount of mental health providers to serve residents in need (500 residents to every 1 mental health provider). Additionally, 27% of Mississippi’s children live in poverty, with 80 child deaths per 100,000 children recorded between 2017 and 2020, which is 30 deaths more than the national average [33].

### 2.2. Data Collection and Sample

Participants included in this study were trainees who completed in-person QPR gatekeeper trainings delivered in tribal schools and MBCI youth-serving organizations. Pre-training and post-training survey responses from CYRI-led training sessions conducted between 2021 and 2023 were analyzed for this study. The pre-training survey consisted of four sociodemographic items (age, gender, race/ethnicity, and education), and nine knowledge and skill items pertaining to suicide. The use of race/ethnicity, rather than singular race or ethnicity, is a direct reflection of the validated survey, such that the standardized instrument was made available to us by QPR. The post-training survey consisted of the same knowledge and skill items as featured in the pre-training survey with two additional training satisfaction items and one open-ended response item for additional comments on the training. (The post-only items are not analyzed in this study, but results are available by request.) In total, 816 completed surveys were submitted to project evaluators from CYRI project leadership. The majority of participating trainees identified as female (*n* = 614, 75.2%), with a smaller number of participants identifying as male (*n* = 165, 20.2%). More than half of all participants were Native American (*n* = 443, 54.3%), followed by whites (*n* = 279, 34.2%), and trainees indicating another race (*n* = 57, 6.9%). The average age of participants was 44.15 years (SD = 11.798) and the average highest education received was about two years of college (4.29 average on scale from 1–6, SD = 1.492). For a breakdown of all sociodemographic characteristics included in the pre-training survey, see Table 1.

### 2.3. Analytical Strategies

To evaluate the effectiveness of QPR gatekeeper training for suicide prevention, we proposed and implemented two analytical strategies. First, we conducted a series of paired-sample *t*-tests to compare differences in pre–post training average scores among trainees in their knowledge gains and skill development/improvement pertinent to suicide and suicide prevention. That is, unadjusted pre–post mean scores were statistically compared. If the *p*-value is ≤0.05, the unadjusted mean differences are deemed statistically significant (minimal likelihood of being due to chance). However, this bivariate analytical strategy is subject to the effects of confounding factors, such as the trainee’s sociodemographic characteristics, including gender, racial/ethnic background, age, and educational attainment. To rule out such potential confounding effects, we implemented our second analytical strategy, namely, panel regression models, specifically, random effects models. These regression models allow us to assess whether post-training is statistically different from pre-training after controlling for gender, racial/ethnic background, age, and educational attainment which are time-invariant covariates. Therefore, if the *p*-value is ≤0.05, the adjusted differences between pre–post-training are deemed statistically significant. Stata Statistical Software Release 18 was used for data analysis [34].

## 3. Results

Turning to the results, Table 2 presents the percentage of respondents for each knowledge and perception-based survey item featured in both the pre-training (pre-test) and post-training (post-test) surveys. The pre-test and post-test surveys are available online (see https://qprinstitute.com/uploads/instructor/Gatekeeper-pre-post-survey.pdf (accessed on 7 February 2024) for the pre-test and post-test surveys). Knowledge and skill ratings included response options of “Low”, “Medium”, and “High”, which were respectively coded 1, 2, and 3. Two items gauging suicide-related perceptions of intent to intervene (items 7 and 8 in Table 2) featured responses positioned in the survey from left to right as “Always”, “Sometimes”, and “Never”. These two items position the most desirable response on the left (first) in the row of response options. Therefore, these items were reverse-coded as “Never” (1), “Sometimes” (2), and “Always” (3) to match the logic of higher scores indicating more salutary responses as found on the questions that precede them (items 1–6 in Table 2). One final item again used a “Low”, “Medium”, and “High” series of possible responses, coded identically to items 1–6. The results for all items in Table 2 present both the pre-training and post-training surveys as statistically significant (<0.001). Utilizing the mean scores, our analysis indicates a general knowledge growth from the pre- to post-training surveys. 

Trainees indicated a relatively low knowledge of facts concerning suicide prevention (mean score = 1.98), warning signs of suicide (1.97), and how to ask about suicide (1.73) on the pre-training survey. Similarly, on the pre-training survey, as expected, trainees generally lacked knowledge on information about local suicide-related resources (1.88). However, relatively greater levels of trainees’ pre-training knowledge of how to persuade someone to get help (2.05) and how to get help for someone themselves (2.05) were evident. When asked about the appropriateness of asking someone about suicidal ideation or intent, trainees mostly indicated sometimes or always, with only a minority noting never (2.16). Trainees also noted on the pre-training survey that they were generally likely to ask someone if they were thinking about suicide, with only a small group unlikely to inquire about suicide (2.03).

The majority of trainees displayed strong evidence of pre-to-post knowledge and skill acquisition across items 1–9 at training completion, with mean scores between 2.33 and 2.75 on the post-training survey (see Table 2). Pre-to-post increases in suicide prevention knowledge and skills were evident in knowing facts concerning suicide (+0.70 pre-to-post mean change), identifying warning signs of suicide (+0.72), knowing how to ask someone about suicide (+0.89), persuading someone to seek help (+0.65), knowing how to get help for someone else (+0.70), and having information about local resources for suicide-related help (+0.83). All of these changes achieved the highest threshold of statistical significance (*p* < 0.001), which indicates an extremely low probability of being due to chance. Subjective trainee confidence in the ability to apply essential skills was also noted in pre-to-post survey comparisons. As expected, the perceived appropriateness of trainees’ asking someone about suicide exhibited a pre-to-post increase (+0.19). Similarly, trainees’ likelihood of asking someone about suicidal thoughts increased from 2.03 on the pre-training survey to 2.33 on the post-training survey, resulting in a pre-to-post difference of +0.3. Moreover, the level of understanding about suicide and suicide prevention increased notably from 2.01 pre to 2.67 post (a difference of +0.66). All of these changes were also statistically significant. These pre-to-post differences and the statistical significance thresholds persisted net of trainee sociodemographic characteristics that are controlled in the random effects models (right-most column in Table 2). In other words, the program was highly effective regardless of trainee race, gender, age, etc. 

## 4. Discussion

Gatekeeper suicide prevention training has been a highly effective method for addressing youth suicide throughout professional and educational contexts. There is substantive research supporting the causal link between gatekeeper training and reduced suicide rates [22]. It is worth noting that suicide rate reductions often result from a combination of factors (i.e., training design, definitions of “gatekeepers”, religious or spiritual beliefs, and systems of support). It is more precise to identify gatekeeper training as improving people’s knowledge, skills, and confidence in helping suicidal individuals [23]. The training also enhances positive beliefs about the efficacy of suicide prevention. Specific elements within the training, such as mental health information provided through lectures, discussions, role-play, and online modules, increase positive training outcomes for trainees of varying professional backgrounds [24]. The purpose of our current study was to shed light on suicide prevention training’s outcomes in Native American populations, an underserved and under-researched group. Therefore, our current study analyzed the effectiveness of QPR gatekeeper training in Mississippi tribal educational and youth-oriented settings. Only one other study has analyzed QPR gatekeeper training efforts in a Native American setting, with similar results [29]. Overall, the analysis of the pre-training and post-training surveys resulted in uniform and statistically significant increases in the knowledge of suicide factors and suicide prevention tools and techniques.

The Mississippi Band of Choctaw Indians (MBCI), through CYRI implementation, have made tangible efforts to increase suicide awareness and prevention efforts, especially pertaining to youth who are most at risk. The introduction of QPR suicide prevention training has yielded strong results, with some areas for improvement especially in the evaluation instrument. Our results indicate the increased knowledge of how to approach and interact with suicidal individuals. An increase in the knowledge of available local and national resources to aid suicidal individuals was also noted. Further, trainees reported having a greater understanding of suicide in general and suicide prevention after training completion. These findings support the current literature that promotes evidence-based gatekeeper training like QPR in professional and educational settings [16,17,18,19].

Experiences with suicide prior to QPR training and feelings efficacy in helping a suicidal youth may also influence post-training outcomes. Trainees who have prior associations with completed or attempted suicides may not have the same confidence levels of those who have encountered positive outcomes with suicidal individuals. Friends and families who care for suicidal individuals show increased self-efficacy in helping during a suicidal event and willingness to do so after gatekeeper training completion [35]. Opportunities for the discussion and practice of QPR techniques during training may increase confidence levels just as they increase knowledge, skills, and awareness. The influence of contextual factors, like the training environment and trainer attitudes, interactions, and speech, can also negatively or positively influence trainee reception to and application of training materials. Addressing ideological beliefs about suicide and individual control or influence over suicidal behaviors can improve long-term outcomes [36]. Integrating socio-cultural aspects of the trainees’ community can also improve the long-term perceptions and knowledge applications of trainees. 

### Limitations and Implications for Future Practice

Our study introduces a replicable model for tribes to integrate into their suicide prevention strategies. While our study analyzed a statistically sound dataset, there are a few limitations. Although the items featured on the standardized survey operated as expected, two comments bear mentioning. First, two of the pre-test/post-test items (items 7 and 8 in Table 2) change the response option logic by featuring the most desirable response option (“Always”) first in the row of possible responses, whereas all other items lead with the least desirable response option (“Low”). No problems were evident in this study, but the switch in the response order could be mentioned to trainees as they move to complete the survey. This revised response order would ensure any misunderstandings are avoided. Individual trainee characteristics (self-confidence in applying suicide prevention techniques), environmental attitudes (cultural stigma towards suicide), and trainer or training material presentation (wording, behavior, approachability, and personability) may create an intersecting barrier against knowledge and skill growth [30]. Second, items 7 and 8 (Table 2) potentially invite generalizations of asking about suicide in any context, which is not always appropriate or necessary. The first of these items, “Do you feel that asking someone about suicide is appropriate?” with response options of always, sometimes, and never, could be revised to reflect contextual specifics, such as “Do you feel that asking someone about suicide is appropriate if they exhibit signs of suicide risk?” The second item, “Do you feel likely to ask someone if they are thinking of suicide?” with the same response options as the first item, could also be revised as “Do you feel comfortable asking someone about suicide if they are exhibiting signs of suicide risk?” These item changes would encourage trainees to think about real-world applications for the techniques and strategies they just learned. The new items would further reflect the point of the assessment, which is the trainees’ knowledge of when to ask about suicide and the trainees’ level of comfort addressing suicide. Of course, changing a standardized instrument has its downsides by potentially undermining measurement consistency across a multiyear project. Therefore, the second of our two considerations may not rise to the level of prompting survey revisions. 

Native American (or American Indian, as identified by the U.S. Census) tribes often implement their own mental health and behavioral health governing standards. In the context of our study, effectiveness refers to the increase in knowledge among trainees and not to a reduction in suicides among the population. Prior to analysis, tailoring the training material to Native American culture and available suicide-related data may encourage training implementation in tribe-supported institutions and completion by tribal and affiliated members [12,29]. Additionally, introducing evidence-based gatekeeper trainings to both faculty and students of secondary or higher-learning education would close the gap between youth at risk of suicide and adults who may encounter and guide these youth. This application would also allow insights into youth receptivity and the application of suicide prevention materials alone and in conjunction with trained adults. Further, introducing a more extensive narrative or open-ended response component to the post-test surveys would expand the understanding of best practices and recommended improvements from current trainees who reflect the needs and desires of future trainees. (The post-test featured a small area for such feedback, but more intensive qualitative methods could be useful for discerning best practices and correcting suboptimal techniques [27]). The inclusion of a 3- or 6-month follow-up survey invites potential survey attrition. However, and more importantly, follow-up surveys also assess the long-term post-training effectiveness of materials versus only short-term effectiveness assessments that were explored in this study. 

## 5. Conclusions

Suicide was the second leading cause of death for youth and young adults aged 10–24 in 2021 [1,2,3,4]. Moreover, suicide rates increased significantly from 2020 to 2021 for non-Hispanic American Indian or Alaska Native, Black, and White males [7,8]. As such, suicide disproportionately affects Native American youth and young adults at higher rates than nearly any other ethnic group in the United States. The implementation of QPR training among Native Americans offers a unique opportunity for professionals of all educational backgrounds to work with youth to expand their knowledge of suicide prevention and prepare them for real-world applications. Though the QPR gatekeeper suicide prevention training program has been previously assessed for effectiveness, our study evaluated QPR training conducted in Mississippi Band of Choctaw Indians-affiliated schools and youth programs as part of the SAMHSA-funded Choctaw Youth Resilience Initiative between 2020 and 2023. QPR has resulted in national and international success through gatekeeper trainees’ growth in suicide prevention knowledge and intended actions before and after training. Our results reveal similar if not greater successes in trainees’ grasp of suicide prevention training materials and willingness to intervene with suicidal youth. The positive and robust results observed here suggests that the existing base of evidence supporting QPR’s suicide prevention gatekeeper program can be successfully implemented for underserved minority populations. The results especially support Native American communities’ receptivity to suicide prevention training in predominantly rural states where suicide is a persistent and leading cause of mortality.

## Figures and Tables

**Table 1:** Sample characteristics (*N* = 816).

Variables	*N*	Percent	Mean	Standard Deviation
Gender				
Male	165	20.2		
Female	614	75.2		
Missing	37	4.5		
Race/ethnicity				
Native American	443	54.3		
White	279	34.2		
Other race	57	6.9		
Missing	37	4.5		
Age			44.15	11.798
Education			4.29	1.492

**Table 2:** Pre–post training analysis: paired-sample *t*-test and panel regression models.

Items	Pre-Training	Post-Training	Paired-Sample *t*-Test	Random Effects Models ^1^
	Mean (95% CI)	Mean (95% CI)	*p*-Value (*n*)	*p*-Value (*n*)
1. Facts concerning suicide prevention	1.98 (1.93–2.04)	2.68 (2.64–2.72)	<0.001 (*n* = 778)	<0.000 (*n* = 763)
2. Warning signs of suicide	1.97 (1.92–2.02)	2.69 (2.65–2.72)	<0.001 (*n* = 781)	<0.001 (*n* = 765)
3. How to ask someone about suicide	1.73 (1.68–1.79)	2.62 (2.58–2.66)	<0.001 (*n* = 780)	<0.000 (*n* = 765)
4. Persuading someone to get help	2.05 (1.99–2.10)	2.70 (2.67–2.74)	<0.001 (*n* = 780)	<0.000 (*n* = 765)
5. How to get help for someone	2.05 (2.00–2.10)	2.75 (2.71–2.78)	<0.001 (*n* = 782)	<0.000 (*n* = 765)
6. Information about local resources for help with suicide	1.88 (1.83–1.92)	2.71 (2.67–2.75)	<0.001 (*n* = 778)	<0.000 (*n* = 765)
7. Do you feel that asking someone about suicide is appropriate?	2.16 (2.12–2.22)	2.35 (2.29–2.40)	<0.001 (*n* = 776)	<0.000 (*n* = 766)
8. Do you feel likely to ask someone if they are thinking of suicide?	2.03 (1.97–2.07)	2.33 (2.27–2.39)	<0.001 (*n* = 779)	<0.000 (*n* = 765)
9. Please rate your level of understanding about suicide and suicide prevention	2.01 (1.96–2.06)	2.67 (2.64–2.70)	<0.001 (*n* = 778)	<0.000 (*n* = 766)

^1^ Time-invariant covariates are gender, race, age, and education.

## Data Availability

The data used to conduct this evaluation are proprietary and not suitable for public release. Please contact the lead author for more information about the data used to conduct this study.
